# Identifying High‐Risk Medications for Drug‐Induced Dystonia: A 20‐Year Retrospective Real‐World Pharmacovigilance Study Based on FAERS

**DOI:** 10.1002/hsr2.72194

**Published:** 2026-04-15

**Authors:** Chunhua Chen, Xiaobin Lin, Yi Yang, Jiajia Yan, Jingxiu Chen, Yifan Zheng, Jia Li

**Affiliations:** ^1^ Department of Pharmacy The First Affiliated Hospital of Sun Yat‐sen University Guangzhou China; ^2^ Department of Pharmacy, Guangdong Provincial People's Hospital Guangdong Academy of Medical Sciences Guangzhou China; ^3^ Department of Clinical Pharmacy Translational Science University of Michigan College of Pharmacy Ann Arbor Michigan USA; ^4^ Department of Pharmacy, Guangxi Hospital Division of The First Affiliated Hospital Sun Yat‐sen University Nanning China

**Keywords:** adverse events, dystonia, FAERS database, pharmacovigilance, time to event onset

## Abstract

**Background and Aims:**

Drug‐induced dystonia is a serious, potentially disabling adverse event (AE) associated with certain medications. Despite its clinical relevance, the existing literature is largely limited to case reports or analyses of individual drugs, and a systematic evaluation of medications implicated in dystonia remains lacking. This study undertook one of the first comprehensive screenings and risk signal rankings of drugs linked to dystonia using data from the FDA adverse event reporting system (FAERS), aiming to inform clinical drug safety.

**Methods:**

Reports of dystonia‐related AEs from Q1 2004 to Q3 2024 were retrieved from FAERS using standardized MedDRA queries. Disproportionality analyses were conducted using the reporting odds ratio (ROR), proportional reporting ratio (PRR), Bayesian Confidence Propagation Neural Network (BCPNN), and Multi‐item Gamma Poisson Shrinker (MGPS) methods to identify potential drug–dystonia signals. The Kaplan–Meier method was applied to assess the time to dystonia onset after drug exposure. Sensitivity analyses using the *Ω* shrinkage estimator were performed to explore potential drug–drug interactions.

**Results:**

A total of 27,618 patients with 28,938 dystonia reports were included. Metoclopramide (7178 reports) and aripiprazole (1595 reports) were most frequently reported. Among the top 50 drugs, metoclopramide showed the strongest disproportionality signal, followed by prochlorperazine and haloperidol. Notably, dystonia was not listed in the package inserts of eight identified drugs, including certain antiepileptics, antidepressants, and antiparkinsonian agents. Most events occurred within 0–30 days of drug initiation. The combination of aripiprazole and risperidone was frequently reported and showed notable interaction signals.

**Conclusions:**

Metoclopramide and several antipsychotics were strongly associated with reported drug‐induced dystonia. These findings highlight the need for cautious prescribing and close monitoring, particularly with antipsychotic combinations, and may support improved risk awareness and labeling.

## 1 Introduction

Dystonia is a complex movement disorder distinguished by sustained or intermittent muscle contractions, which result in abnormal and often repetitive movements, postures, or a combination of both [1, 2]. Research indicates that the global prevalence of dystonia is approximately 0.06% [3], with women constitute 60% to 70% of those affected [3, 4]. The highest prevalence is found in white populations. Black, Asian, and mixed‐race populations show significantly lower rates [3]. Involuntary muscle contractions can lead to abnormal movements and postures, and nearly 95% of severe cases result in an inability to perform self‐care tasks or work [1, 5, 6]. Some may also develop severe depression and/or anxiety [7].

Dystonia is widely recognized as a multifactorial condition influenced by genetic factors, brain injuries, and drug adverse effects [8, 9]. Drug‐induced dystonia is generally divided into acute and tardive dystonia depending on the time of onset, and can include the neck (torticollis), hands (writing spasms), or mouth (oromandibular dystonia) depending on the site of involvement [10, 11]. Drug‐induced dystonia, as a subtype, has a relatively low prevalence. In a closed cohort study involving 525,731 individuals, the point prevalence of drug‐induced tardive dystonia was estimated at approximately 0.004% [4]. Among drugs that induce dystonia, antipsychotics account for 1.4% to 16.5%, while metoclopramide accounts for 0.2% [12]. It is crucial to clarify the difference between drug‐induced dystonia, which is an adverse event (AE), and dystonia, in terms of the duration and severity of the symptoms experienced.

However, despite the potential for serious clinical consequences, public awareness of drug‐induced dystonia remains limited [13]. Previous research has focused predominantly on antipsychotic agents, while studies investigating dystonia‐related AEs induced by other potentially relevant medications (e.g., propofol, ondansetron) are relatively scarce [14, 15, 16]. Although drug‐induced dystonia may pose a notable threat to patient health, its precise precipitating factors and underlying mechanisms have not been identified. In parallel with the gradual introduction of newer antipsychotics, such as pimavanserin and lumateperone, their safety profiles, particularly with regard to dystonia risk, have yet to be fully characterized [17, 18]. In recent years, few studies have assessed drug‐induced dystonia. Further epidemiological studies are necessary to identify and prevent safety issues and AEs associated with drug‐induced dystonia, increase public awareness, and mitigate risks.

Our study used a real‐world population dataset from the Food and Drug Administration (FDA) Adverse Event Reporting System (FAERS) database, which contains extensive data on patient AEs. This database is critical to supporting the FDA's postmarketing surveillance of drugs and therapeutic biologics. It facilitates the mining and quantitative analysis of adverse drug reactions, thus aiding in the safe clinical use of medications. Additionally, it enables the identification of emerging risks associated with numerous new drugs [19]. To our knowledge, few FAERS databases have specifically included dystonia in conjunction with reported drug associations. The aim of this study was to use FAERS to identify drug‐related precursors of dystonia and assess the risk profiles of drug combinations, to aid in clinical decision‐making and patient management.

## 2 Methods

### 2.1 Data Source and Collection

ASCII data packets were downloaded from the FAERS database on the FDA website (https://fis.fda.gov/extensions/FPD-QDE-FAERS/FPD-QDE-FAERS.html) for the period from Q1 2004 to Q3 2024, and were analyzed in SAS 9.4 software after data cleaning. Duplicate data were removed, in accordance with the official guidance documents recommended by the FDA [20]. We extracted the “PRIMARYID,” “CASEID,” and “FDA_DT” fields from the “DEMO” table and sorted them in descending order by “CASEID,” “FDA_DT,” and “PRIMARYID.” For records with identical CASEID values, only the record with the largest FDA_DT value was retained. If both the CASEID and FDA_DT values matched, the record with the largest PRIMARYID value was kept. As each quarterly data package from Q1 2019 onwards included a deletion report list, the corresponding reports were removed based on the CASEID values in the list after completing the above deduplication.

### 2.2 Definition of AEs and Drugs

AEs were classified according to preferred terms (PTs) from the Medical Dictionary for Regulatory Activities (MedDRA), version 27.1. The Standardized MedDRA Query (SMQ) broad definition search strategy identified 42 PTs related to “dystonia”, whereas the SMQ narrow definition strategy identified 14 PTs (see Supporting Information: Table S1 for details). To improve specificity and minimize potential misclassification from loosely related terms, we used the SMQ narrow definition approach to focus on the core clinical manifestations of dystonia. Adverse drug event (ADE) reports were retrieved according to these PTs, and potential drugs were identified. The analysis was restricted to the drugs identified as having the highest suspicion for inducing dystonia. The drugs' names in the database were standardized according to the World Health Organization Drug Dictionary (September 2024) and analyzed according to the anatomical therapeutic chemical (ATC) classification and clinical use.

### 2.3 Statistical Analysis

Descriptive analysis included the general information in ADE reports associated with drug‐induced dystonia, including patient age, sex, outcome, reporter, reporting year, reporting country, and timing of adverse reaction onset. The reporting odds ratio (ROR), proportional reporting ratio (PRR), Bayesian confidence propagation neural network (BCPNN), and multi‐item gamma Poisson shrinker (MGPS) methods were applied in the disproportionate analysis for positive signal calculation [21, 22, 23].

When the lower bound of the 95% confidence interval (CI) exceeds 1, the ROR has been shown to provide an appropriate balance between sensitivity and statistical reliability, making it suitable for the early identification of potential drug–event associations [24]. The PRR thresholds applied in this study are consistent with European Medicines Agency (EMA) recommendations and established field consensus, requiring a minimum of three reports, a PRR ≥ 2, and a *χ*² > 4 [25]. These criteria aim to retain sufficient sensitivity for signal detection while limiting the risk of spurious associations. For the BCPNN method, a minimum of three reports in combination with an IC_025_ > 0 was required to reduce the influence of isolated reports and random variation while maintaining statistical reliability [26]. To further differentiate weak or uncertain associations from more robust signals, the lower 90% confidence limit of the empirical Bayes geometric mean (EBGM05) was set at > 2, a threshold commonly used to improve signal reliability in pharmacovigilance analyses [24]. Finally, to strengthen the overall robustness of the findings, we adopted a deliberately conservative definition of a positive signal. A drug–event pair was retained only when all four algorithms simultaneously met their respective criteria (Table 1). This approach reduces variability arising from method‐specific assumptions, substantially lowers the likelihood of false‐positive findings, and mitigates inconsistencies across different signal detection methods.

**Table 1:** Formulas and signal principles of the four main signal detection and sensitivity analysis algorithms.

Algorithms	Formulas	Threshold
ROR	ROR $=\frac{a/c}{b/d}$	*a* ≥ 3, lower limit of ROR_95%CI_ > 1
	${95\%\text{CI}=e}^{\ln\left(\mathrm{ROR}\right)\pm 1.96\sqrt{\frac{1}{a}+\frac{1}{b}+\frac{1}{c}+\frac{1}{d}}}$	
PRR	PRR $=\frac{a/(a+b)}{c/(c+d)}$	*a* ≥ 3, PRR ≥ 2, *χ* ^2^ ≥ 4
	$\chi 2=\frac{\left(a+b+c+d\right){\left(ad-\mathrm{bc}\right)}^{2}}{\left(a+b\right)(c+d)(a+c)(b+d)}$	
BCPNN	$IC=\log_{2}\frac{a+0.5}{a_{\text{exp}}+0.5}$	*a* ≥ 3, IC_025_ > 0
	$a_{\text{exp}}=\frac{\left(a+b)(a+c\right)}{a+b+c+d}$	
	${\mathrm{IC}}_{025}=IC-3.3{\left(a+0.5\right)}^{-0.5}-2{\left(a+0.5\right)}^{-1.5}$	
MGPS	$\mathrm{EBGM}=\frac{a(a+b+c+d)}{\left(a+c)(a+b\right)}$	a ≥ 3, EBGM_05_ > 2
	${\mathrm{EBGM}}_{05}{=e}^{\ln(\mathrm{EGBM})}-1.64{\left(\frac{1}{a}+\frac{1}{b}+\frac{1}{c}+\frac{1}{d}\right)}^{-0.5}$	
*Ω* shrinkage measure model	$\Omega =\log_{2}\frac{n111+0.5}{E111+0.5}$	*Ω*_025_ > 0
	$\Omega_{025}=\Omega -1.96\sqrt{\log_{2}\frac{n111+0.5}{E111+0.5}}$	

Abbreviations: a, number of reports including both the target drug and target adverse drug reaction; b, number of reports including other adverse drug reactions for the target drug; c, number of reports including target adverse drug reactions for other drugs; CI, confidence interval; *χ*, chi‐square test*^2^*; d, number of reports including other drugs and adverse drug reactions; EBGM, empirical Bayesian geometric mean; *E111*, expected value of adverse events targeted by combinations of two drugs; EGBM*05,* lower limit of the 95% CI of EBGM; IC, information component; IC_025_, lower limit of 95% CI of the IC; *n111*, reported number of adverse events targeted by combinations of two drugs; *Ω*_*025*_, lower limit of the 95% CI of *Ω*.

Time to onset (TTO) was defined as the interval between drug initiation and the occurrence of the AE. Reports with incomplete, implausible, or inconsistent date information were excluded to ensure data accuracy. The TTO data were summarized using the median and interquartile range (IQR). All statistical tests were two‐sided, and *p* < 0.05 was considered statistically significant [27]. We used the Kaplan–Meier method to estimate the cumulative percentage of drug‐induced dystonia in the various drug treatment groups, and displayed the probability trend of AEs occurring in different groups over time. The Log‐rank Test was used as the formal inference (significance level *α* = 0.05), with the results of the uncensored Kruskal–Wallis Test supplemented to visualize raw time distribution. Notably, the Kruskal–Wallis Test serves only to describe the raw time distribution and not for statistical inference. The *Ω* shrinkage measure model (Table 1), a statistical method based on the ratio of observed reports to expected values, was used for signal detection of AEs [28]. A notable characteristic of this model is its relative conservatism during the detection process, thus enhancing the precision of signal detection. A lower limit of *Ω*_025_ degrees indicates the presence of drug‐drug interactions [23]. This method can be used in combination with other methods to consider the interference of drug interaction factors and screen for drug‐drug combinations that increase the risk of target ADE occurrence. All the above statistical analyses were performed using SAS 9.4 software.

### 2.4 Ethical Considerations

This study was based exclusively on data obtained from the FAERS, a publicly accessible database containing de‐identified and anonymized reports. No individual‐level identifiable information was used, and no direct interaction with human participants occurred. Therefore, ethical approval and informed consent were not required for this study in accordance with applicable regulations and institutional guidelines.

## 3 Results

### 3.1 Descriptive Analysis

A total of 21,964,449 patient reports were retrieved from the FAERS database, and 3,686,206 duplicate reports for the same patients were removed. Further screening yielded 28,938 reports of dystonia in 27,618 patients. From 2004 to 2024, a general trend indicating fluctuating growth in dystonia reports, which peaked in 2011 and 2012, with 4270 and 4273 cases reported in those years, respectively (Supporting Information: Figure S1).

Table 2 summarizes the clinical features of these reports. Female patients (54.84%) experienced dystonia at a rate nearly 1.5 times that of male patients (35.98%). The highest prevalence of dystonia AEs was observed in patients 18–44 years of age (21.11%), followed by those 45–64 years of age (13.01%). Lawyers reported a relatively high rate of 24.87% AEs, and were followed by physicians (21.94%) and consumers (21.25%). The United States (56.12%) reported more than half of the drug‐related dystonia AE cases, accounting for substantially more cases than observed in the United Kingdom (7.85%), Canada (4.61%), Italy (2.80%), and Germany (2.77%). Serious AE reports included prolonged hospitalization (32.86%), disabilities (25.61%), life‐threatening events (3.71%), or death (2.87%). The most common indication for patients was abdominal distension/dyspepsia, in 1930 patients (Supporting Information: Table S2).

**Table 2:** Characteristics of drug‐induced dystonia reports.

Characteristics	Number of cases, *n* (%)
**Sex**	
Female	15,146 (54.84)
Male	9936 (35.98)
Not specified	2536 (9.18)
**Age (years)**	
< 18	2907 (10.53)
18–44	5829 (21.11)
45–64	3592 (13.01)
65–74	1647 (5.96)
75–84	960 (3.48)
≥ 85	261 (0.95)
Unknown	12,422 (44.98)
**Reporter**	
Lawyer	6869 (24.87)
Physician	6060 (21.94)
Consumer	5868 (21.25)
Other health‐professional	4112 (14.89)
Pharmacist	3641 (13.18)
Not specified	1068 (3.87)
**Top five reporting countries**	
United States of America	15,500 (56.12)
United Kingdom	2168 (7.85)
Canada	1274 (4.61)
Italy	773 (2.80)
Germany	766 (2.77)
**Outcome**	
Hospitalization: initial or prolonged	9075 (32.86)
Disability	7074 (25.61)
Life‐threatening	1026 (3.71)
Death	792 (2.87)
Required intervention to prevent permanent Impairment/damage	334 (1.21)
Congenital anomaly	250 (0.91)
Other	17,146 (62.08)
**Time to event onset (days)**	
*N* (Missing)	5657 (21,961)
Mean (SD)	270.61 (814.00)
Median (Q1, Q3)	5.00 (0.00,91.00)
Min, Max	0.00, 12387.00

### 3.2 Disproportionality Analysis

**Figure 1:**
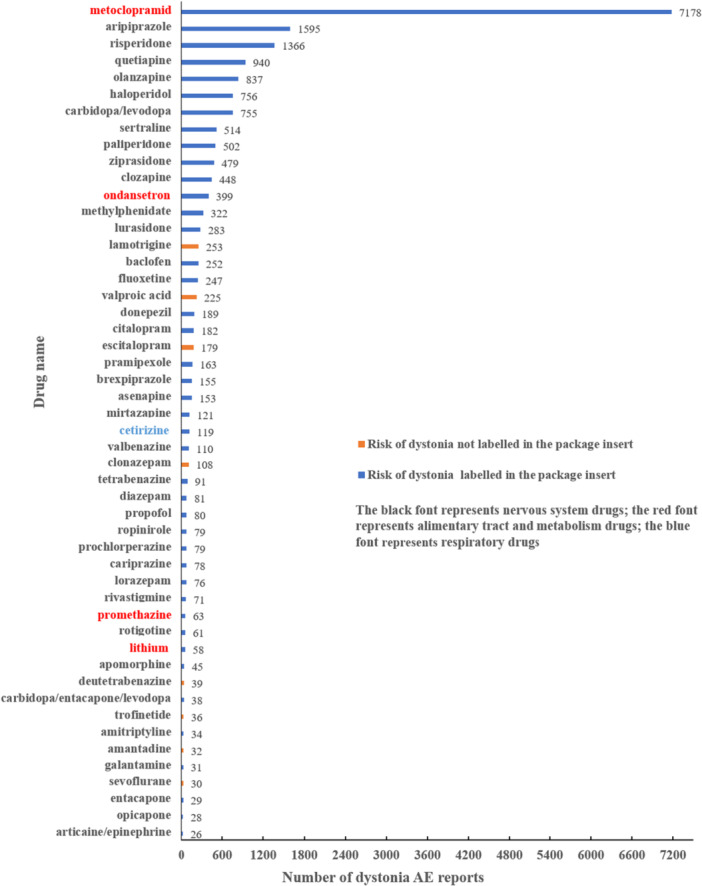
Top 50 drugs with dystonia reports, ranked by case number.

Figure 1 shows the number of the top 50 drugs (ranked by case number) among those with positive signals for drug‐induced dystonia. Metoclopramide had the highest number of reports for drug‐induced dystonia (7178 reports), and was followed by aripiprazole (1595 reports), risperidone (1366 reports), quetiapine (940 reports), olanzapine (837 reports), haloperidol (756 reports), carbidopa/levodopa (755 reports), sertraline (514 reports), paliperidone (502 reports), and ziprasidone (479 reports). Among the top 50 drugs identified, 45 were nervous system drugs, four were alimentary tract and metabolism drugs, and only one was a respiratory system drug, according to the ATC classification system. The risk of dystonia was not included in the package labeling for the following eight drugs: lamotrigine, valproic acid, escitalopram, clonazepam, deutetrabenazine, trofinetide, amantadine, and sevoflurane.

Table 3 presents the signal strength of the top 50 drugs, ordered by the number of reported cases. Drugs were included if they met two criteria: the presence of a positive signal for drug‐induced dystonia and a relatively high reporting frequency among signal‐positive agents. Metoclopramide (ROR [95% CI], 155.90 [151.70, 160.21], PRR [χ^2^], 146.77 [781861.00]) showed the most pronounced signal values, and was followed by prochlorperazine (ROR [95% CI], 76.75 [61.27, 96.14], PRR [χ^2^], 73.78 [5659.16]), haloperidol (ROR [95% CI], 37.26 [34.64, 40.08], PRR [χ^2^], 36.57 [25485.30]), ziprasidone (ROR [95% CI], 28.99 [26.47, 31.75], PRR [χ^2^], 28.57 [12541.30]), opicapone (ROR [95% CI], 26.19 [18.04, 38.04], PRR [χ^2^], 25.85 [668.51]), asenapine (ROR [95% CI], 16.97 [14.47, 19.91], PRR [χ^2^], 16.83 [2266.84]), aripiprazole (ROR [95% CI], 14.73 [14.00, 15.49], PRR [χ^2^], 14.62 [19139.10]), cariprazine (ROR [95% CI], 13.97 [11.18, 17.47], PRR [χ^2^], 13.88 [930.18]), donepezil (ROR [95% CI], 13.80 [11.96, 15.93], PRR [χ^2^], 13.71 [2213.59]), and lurasidone (ROR [95% CI], 13.08 [11.63, 14.71], PRR [χ^2^], 13.00 [3105.45]).

**Table 3:** Top 50 drugs associated with dystonia based on reporting frequency (ranked by descending number of reported cases).

Drug name	Case reports	ROR (95% CI)	PRR (*χ* ^2^)	IC (IC_025_)	EBGM (EBGM_05_)
Metoclopramideª	7178	155.90 (151.70, 160.21)	146.77 (781861.00)	6.79 (6.73)	110.61 (107.63)
Aripiprazoleⁿ	1595	14.73 (14.00, 15.49)	14.62 (19139.10)	3.79 (3.71)	13.87 (13.19)
Risperidoneⁿ	1366	11.85 (11.22, 12.51)	11.78 (12848.40)	3.49 (3.40)	11.27 (10.67)
Quetiapineⁿ	940	6.12 (5.73, 6.53)	6.10 (3883.06)	2.57 (2.47)	5.94 (5.56)
Olanzapineⁿ	837	8.55 (7.98, 9.16)	8.52 (5396.50)	3.05 (2.94)	8.30 (7.75)
Haloperidolⁿ	756	37.26 (34.64, 40.08)	36.57 (25485.30)	5.16 (4.98)	35.64 (33.13)
Carbidopa/levodopaⁿ	755	6.75 (6.28, 7.26)	6.73 (3591.79)	2.72 (2.60)	6.58 (6.12)
Sertralineⁿ	514	5.27 (4.83, 5.75)	5.26 (1742.19)	2.37 (2.23)	5.18 (4.75)
Paliperidoneⁿ	502	11.44 (10.47, 12.50)	11.38 (4672.36)	3.49 (3.33)	11.20 (10.25)
Ziprasidoneⁿ	479	28.99 (26.47, 31.75)	28.57 (12541.30)	4.81 (4.60)	28.12 (25.67)
Clozapineⁿ	448	3.27 (2.98, 3.59)	3.26 (692.84)	1.69 (1.55)	3.23 (2.94)
Ondansetronª	399	10.65 (9.65, 11.76)	10.60 (3422.18)	3.39 (3.21)	10.47 (9.48)
Methylphenidateⁿ	322	5.00 (4.48, 5.59)	4.99 (1017.72)	2.31 (2.13)	4.95 (4.43)
Lurasidoneⁿ	283	13.08 (11.63, 14.71)	13.00 (3105.45)	3.69 (3.46)	12.88 (11.45)
Lamotrigineⁿ	253	2.49 (2.20, 2.82)	2.49 (222.92)	1.31 (1.12)	2.47 (2.19)
Baclofenⁿ	252	4.47 (3.95, 5.07)	4.47 (672.29)	2.15 (1.95)	4.44 (3.92)
Fluoxetineⁿ	247	4.83 (4.26, 5.47)	4.82 (741.13)	2.26 (2.05)	4.78 (4.22)
Valproic acidⁿ	225	2.93 (2.57, 3.34)	2.93 (282.99)	1.54 (1.34)	2.91 (2.55)
Donepezilⁿ	189	13.80 (11.96, 15.93)	13.71 (2213.59)	3.77 (3.46)	13.63 (11.80)
Citalopramⁿ	182	3.53 (3.05, 4.09)	3.53 (327.75)	1.81 (1.58)	3.51 (3.04)
Escitalopramⁿ	179	3.94 (3.40, 4.56)	3.93 (389.07)	1.97 (1.73)	3.91 (3.38)
Pramipexoleⁿ	163	9.91 (8.49, 11.56)	9.86 (1291.04)	3.29 (2.99)	9.81 (8.41)
Brexpiprazoleⁿ	155	12.19 (10.41, 14.29)	12.12 (1574.07)	3.59 (3.26)	12.06 (10.30)
Asenapineⁿ	153	16.97 (14.47, 19.91)	16.83 (2266.84)	4.07 (3.69)	16.74 (14.27)
Mirtazapineⁿ	121	3.36 (2.81, 4.02)	3.36 (199.54)	1.74 (1.45)	3.35 (2.80)
Cetirizineʳ	119	2.64 (2.20, 3.16)	2.64 (120.44)	1.39 (1.11)	2.63 (2.20)
Valbenazineⁿ	110	4.94 (4.10, 5.96)	4.93 (343.40)	2.30 (1.97)	4.91 (4.07)
Clonazepamⁿ	108	2.44 (2.02, 2.94)	2.43 (91.00)	1.28 (0.98)	2.43 (2.01)
Tetrabenazineⁿ	91	9.46 (7.69, 11.62)	9.41 (682.45)	3.23 (2.80)	9.39 (7.64)
Diazepamⁿ	81	2.72 (2.19, 3.38)	2.72 (87.78)	1.44 (1.09)	2.71 (2.18)
Propofolⁿ	80	6.79 (5.45, 8.45)	6.76 (392.13)	2.75 (2.33)	6.75 (5.42)
Prochlorperazineⁿ	79	76.75 (61.27, 96.14)	73.78 (5659.16)	6.20 (4.94)	73.58 (58.74)
Ropiniroleⁿ	79	5.99 (4.80, 7.47)	5.97 (326.40)	2.58 (2.16)	5.96 (4.78)
Cariprazineⁿ	78	13.97 (11.18, 17.47)	13.88 (930.18)	3.79 (3.25)	13.84 (11.08)
Lorazepamⁿ	76	2.51 (2.01, 3.15)	2.51 (68.87)	1.33 (0.97)	2.51 (2.00)
Rivastigmineⁿ	71	2.63 (2.08, 3.31)	2.62 (71.18)	1.39 (1.02)	2.62 (2.07)
Promethazineª	63	9.60 (7.50,12.31)	9.56 (482.13)	3.25 (2.71)	9.54 (7.45)
Rotigotineⁿ	61	4.80 (3.73, 6.17)	4.79 (182.53)	2.26 (1.80)	4.78 (3.72)
Lithiumª	58	3.74 (2.89, 4.83)	3.73 (115.74)	1.90 (1.46)	3.72 (2.88)
Apomorphineⁿ	45	3.68 (2.75, 4.93)	3.68 (87.61)	1.88 (1.37)	3.67 (2.74)
Deutetrabenazineⁿ	39	6.95 (5.07, 9.52)	6.93 (197.59)	2.79 (2.13)	6.92 (5.05)
Carbidopa/Entacapone/Levodopaⁿ	38	7.96 (5.78, 10.94)	7.93 (229.83)	2.99 (2.29)	7.92 (5.76)
Trofinetideⁿ	36	5.26 (3.79, 7.30)	5.25 (123.86)	2.39 (1.76)	5.25 (3.78)
Amitriptylineⁿ	34	3.47 (2.47, 4.85)	3.46 (59.44)	1.79 (1.20)	3.46 (2.47)
Amantadineⁿ	32	6.06 (4.29, 8.58)	6.05 (134.75)	2.60 (1.89)	6.04 (4.27)
Galantamineⁿ	31	6.99 (4.91, 9.95)	6.97 (158.46)	2.80 (2.04)	6.96 (4.89)
Sevofluraneⁿ	30	6.10 (4.26, 8.73)	6.08 (127.27)	2.60 (1.86)	6.07 (4.24)
Entacaponeⁿ	29	7.46 (5.18, 10.75)	7.44 (161.56)	2.89 (2.09)	7.43 (5.16)
Opicaponeⁿ	28	26.19 (18.04, 38.04)	25.85 (668.51)	4.69 (3.26)	25.82 (17.78)
Articaine/epinephrineⁿ	26	12.18 (8.28, 17.92)	12.11 (264.89)	3.60 (2.54)	12.10 (8.23)

Abbreviations: CI, confidence interval; EBGM, empirical Bayesian geometric mean; EBGM_05_, lower limit of the 95% CI of EGBM; IC, information component; PRR, proportional reporting ratio; ROR, reporting odds ratio; χ^2^, chi‐square; a, alimentary tract and metabolism; n, nervous system; r, respiratory system.

### 3.3 TTO Analysis

**Figure 2:**
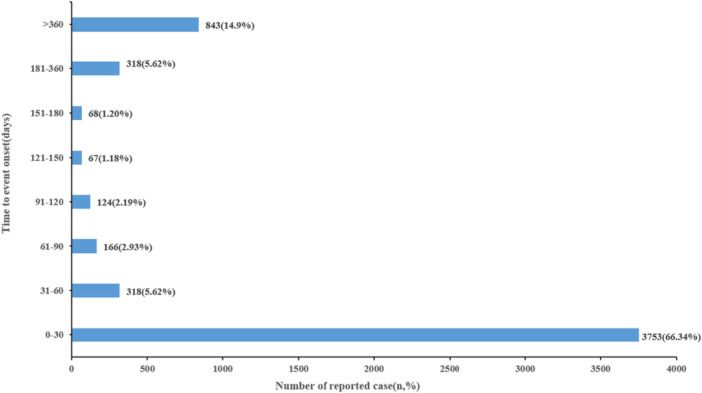
Time to event onset in drug‐induced dystonia cases, reported as number/percentage.

A total of 5657 FAERS reports with complete information on both drug initiation and AE onset were included in the TTO analysis; reports with missing or implausible time data were excluded. The median TTO was 5 days (IQR: Q1 = 0, Q3 = 91) (Table 2). Overall, 66.34% (3753 cases) of dystonia reports occurred within 0–30 days after drug initiation, whereas 14.99% of cases were reported more than 360 days after exposure (Figure 2).

**Figure 3:**
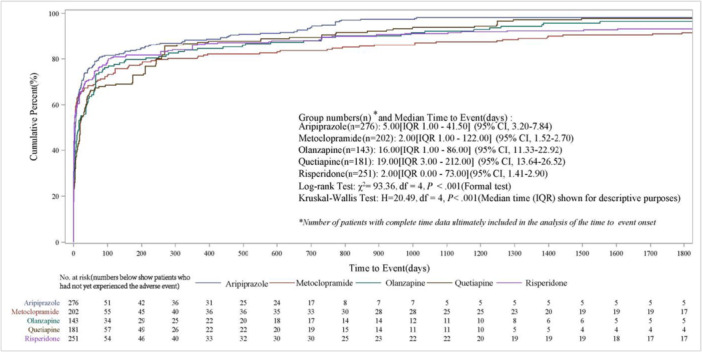
Kaplan–Meier estimates of the time to event and cumulative percentage for the top five drug‐induced dystonia medications.

The top five drugs by number of reports, metoclopramide, aripiprazole, risperidone, quetiapine, and olanzapine, were selected for further analysis. A total of 11,456 reports were identified, including 1506 related to aripiprazole, 7100 to metoclopramide, 767 to olanzapine, 873 to quetiapine, and 1210 to risperidone. Among these, 1053 reports contained complete information on both the start and end dates of the AEs, comprising 276 for aripiprazole, 202 for metoclopramide, 143 for olanzapine, 181 for quetiapine, and 251 for risperidone. Cumulative percentages were analyzed according to onset time. The median time to event following drug administration was 5 days (95% CI, 3.20–7.84) for aripiprazole, 2 days (95% CI, 1.52–2.70) for metoclopramide, 16 days (95% CI, 11.33–22.92) for olanzapine, 19 days (95% CI, 13.64–26.52) for quetiapine, and 2 days (95% CI, 1.41–2.90) for risperidone. Kaplan–Meier analysis demonstrated a significant difference in time to dystonia onset among the five drug groups (log‐rank *χ*² = 93.36, df = 4, *p* < 0.001; Figure 3). This finding was further supported by the Kruskal–Wallis test, which indicated a statistically significant difference in median TTO across groups (*p* < 0.001).

Figure 3 shows the number of cases at risk at each time point. As follow‐up time increased, the number of individuals in each group who had not yet developed dystonia progressively decreased. According to the number‐at‐risk table, after approximately 1300 days of follow‐up, the number of at‐risk patients in most groups had fallen to fewer than 10 individuals. Methodological guidelines indicate that estimates in the tail of Kaplan–Meier curves become increasingly unstable when the number of at‐risk individuals in any group falls below 5–10 [29]. Therefore, findings related to long‐term temporal patterns of AEs (e.g., beyond 1300 days) should be considered exploratory and interpreted with caution.

### 3.4 Drug‐Drug Interaction Analysis

**Figure 4:**
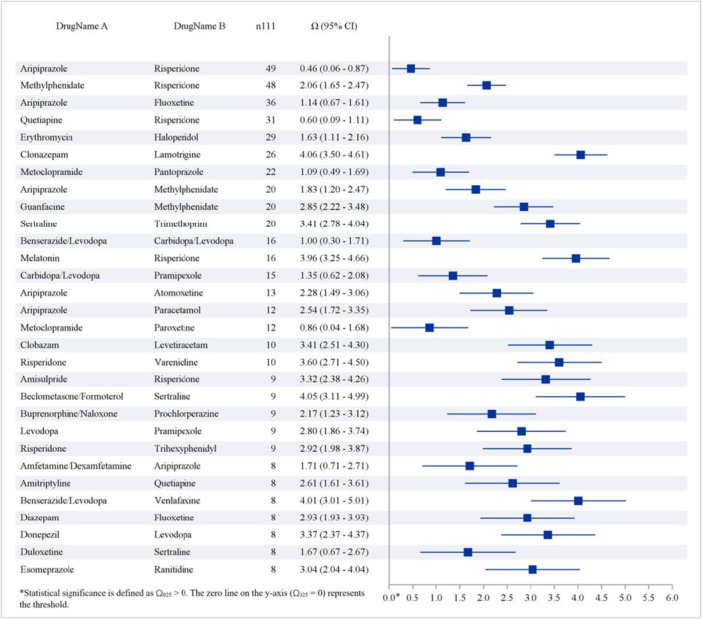
Sensitivity analysis of the top 30 drug‐induced dystonia cases based on drug–drug–adverse event co‐reporting. Legend: This figure presents a sensitivity analysis of the top 30 drug‐induced dystonia cases derived from reports involving two‐drug combinations. The metric *n111* denotes the number of reports in which dystonia was reported following the concomitant use of drug A and drug B. Each bar represents the reporting signal associated with a specific two‐drug combination. Drug combinations are depicted as blue bars; combinations whose confidence intervals (error bars) do not cross the null reference line are considered to show statistically significant disproportionality signals.

The top 30 drug interactions that increased the risk of dystonia are displayed in Figure 4. The combination of aripiprazole with risperidone (n111, 49, Ω [95% CI], 0.46 [0.06–0.87]) had the highest number of related AEs. Other notable combinations included methylphenidate with risperidone (n111, 48, Ω [95% CI], 2.06 [1.65–2.47]), aripiprazole with fluoxetine (n111, 36, Ω [95% CI], 1.14 [0.67–1.61]), quetiapine with risperidone (n111, 31, Ω [95% CI], 0.60 [0.09–1.11]), and erythromycin with haloperidol (n111, 29, Ω [95% CI], 1.63 [1.11–2.61]).

## 4 Discussion

Through a comprehensive pharmacovigilance signal detection approach, this study provides an integrated assessment of drug–dystonia associations using FAERS data collected between 2004 Q1 and 2024 Q3. Overall, most reported clinical outcomes were severe: 32.86% resulted in hospitalization or prolonged length of stay, thus indicating the severity of dystonia as an AE. 96 drugs with positive signals (Supporting Information: Table S3) were identified according to a combination of ROR, PRR, IC_025_, and EBGM_05_. Previous studies have confirmed that antipsychotics were the most common cause [9]. This is consistent with the findings of our research. According to the ATC classification, nervous system drugs, and alimentary tract and metabolism drugs were the two most frequently reported drugs causing dystonia (Supporting Information: Table S4). Studies have shown that dopamine receptor antagonists and metoclopramide induce acute dystonia [9, 30]. In line with this established pharmacological mechanism, metoclopramide was the most frequently reported drug in our analysis, followed by antipsychotic agents, predominantly dopamine *D*₂ receptor antagonists. First‐generation antipsychotics have also been consistently identified as being commonly associated with dystonia‐related AEs in prior investigations [12]. Notably, although metoclopramide accounted for the largest number of dystonia reports in the present study, this pattern is likely influenced by differences in reporting practices and inherent limitations of spontaneous reporting systems, rather than indicating a higher intrinsic risk compared with other agents.

In this analysis, dystonia was reported more frequently in women (15,146 patients) than in men (9936 patients). Previous evidence suggests that oestrogen may modulate dopaminergic neurotransmission [12, 31], which could partly explain sex‐related variability in susceptibility to pharmacological agents and the development of motor adverse effects [31]. Although immunomodulatory drugs had the third highest number of reports (1929 reports), the IC_025_ and EBGM_05_ values did not reach the thresholds. This discrepancy suggests that the observed reports may reflect nonspecific reporting patterns or confounding factors, warranting further investigation to clarify their potential association with dystonia.

The top 10 drugs in terms of the number of reports associated with dystonia, including aripiprazole, risperidone, quetiapine, and haloperidol, all had dystonia‐related AEs reported in the literature [32, 33, 34]. These drugs work by blocking dopamine receptors and are used to treat psychiatric disorders such as schizophrenia. Drug‐induced dystonia incidence has been suggested to be associated with dopamine *D*_2_ receptor affinity, and focal dystonia might potentially stem from misprocessed somatosensory inputs causing erroneous motor program execution [11, 35, 36]. Metoclopramide, a dopamine *D*_2_ receptor antagonist commonly prescribed for nausea, vomiting, and belching, was associated with the largest number of reported dystonia cases and exhibited the strongest ROR signal among the top 50 drugs identified. Prior research has demonstrated that acute dystonia is the most common type of extrapyramidal reaction associated with metoclopramide [37]. In 2009, the FDA issued a black box warning for metoclopramide, stating that it can induce tardive dyskinesia and cause irreversible damage. Moreover, a meta‐analysis demonstrated that a substantial proportion of pediatric patients treated with metoclopramide developed acute focal dystonia, most commonly involving the neck, ocular, and orolingual regions [38].

Among the top 50 drugs with the highest number of reported dystonia cases, the 10 agents with the strongest ROR signals were predominantly antipsychotics, with metoclopramide being the only nonantipsychotic exception. Most of these drugs act as dopamine receptor antagonists, including prochlorperazine and haloperidol, which ranked second and third, respectively. In a controlled trial, patients in the haloperidol group required fewer injections than those in the aripiprazole group but experienced a higher incidence of dystonia [38], a finding that aligns with the stronger risk signal observed for haloperidol in our analysis. In addition to dopamine receptor antagonists, our pharmacovigilance screening identified positive dystonia signals for several selective serotonin reuptake inhibitors (SSRIs), such as sertraline, fluoxetine, and citalopram. Although SSRIs do not directly block dopamine receptors, preclinical and clinical studies suggest that modulation of serotonergic pathways, particularly involving 5‐HT receptors, may indirectly influence dopaminergic neurotransmission, thereby increasing susceptibility to extrapyramidal motor symptoms, including dystonia [13]. Conversely, partial dopamine agonists such as aripiprazole may also precipitate dystonia through a different pharmacodynamic mechanism. By altering dopaminergic tone and receptor signaling balance, these agents may disrupt neuromuscular control, leading to abnormal motor output [13, 39]. Collectively, these findings suggest that both dopamine antagonism and dysregulation of serotonergic–dopaminergic interactions may converge on shared neurobiological pathways underlying drug‐induced dystonia.

Beyond metoclopramide and antipsychotic agents, our pharmacovigilance analysis identified several additional medications showing positive signals for dystonia. Ondansetron, in particular, demonstrated a significant association, in agreement with previously reported clinical cases [16, 40]. Although the biological mechanism underlying this association remains unclear, existing evidence suggests that ondansetron may affect motor control through indirect alterations in dopaminergic neurotransmission or complex interactions among central receptor systems [40]. Furthermore, signals were also observed for nonantipsychotic drug classes, including antiepileptic agents such as lamotrigine and valproic acid, as well as antihistamines such as cetirizine. These findings are consistent with earlier reviews and case‐based observations [41]. Taken together with prior evidence, our results highlight that dystonia may arise in association with a wider range of medications than traditionally recognized, emphasizing the importance of considering these agents when assessing patients with unexplained or atypical dystonic presentations.

Significantly, we found that a substantial proportion of reports in the FAERS database were submitted by legal professionals, raising the possibility of litigation‐driven reporting bias. Such bias has been described as a tendency to disproportionately amplify or selectively report AEs in the context of legal claims [42, 43]. To evaluate the extent to which lawyer‐submitted reports affected signal detection, we performed a subgroup analysis excluding these reports. After removal, the number of drugs meeting positive signal criteria increased from 96 to 111 (Supporting Information: Table S5), suggesting that litigation‐related reporting may obscure weaker but potentially meaningful safety signals. This effect was particularly evident for metoclopramide, for which the number of dystonia reports declined markedly after exclusion, indicating that its reporting volume was largely driven by legal submissions (Supporting Information: Table S6). Importantly, despite these changes in absolute report counts, the overall signal structure remained stable. The top‐ranked drugs showed minimal shifts in relative ranking, and signal strength estimates generally increased following exclusion of lawyer reports, for example, the ROR for aripiprazole rose from 14.73 to 19.59. This pattern suggests that reports from healthcare professionals and other nonlegal sources may carry a higher signal‐to‐noise ratio and clearer clinical attribution. These findings indicate that litigation bias can substantially distort reporting volume for specific drugs while having a more limited impact on the identification of core drug–dystonia associations. Excluding attorney‐reported cases did not materially change the leading signals. Our results underscore the importance of considering reporter composition when interpreting pharmacovigilance signals derived from spontaneous reporting systems.

In parallel with reporter type, geographic concentration also appeared to shape the reporting landscape. Over half of all dystonia reports originated from the United States, a pattern likely reflecting both the size of the United States pharmaceutical market and the legal and regulatory environment surrounding AE reporting. This interpretation is supported by the observation that nearly all lawyer‐submitted reports in our dataset were filed from the United States (Supporting Information: Table S5). Such geographic clustering may contribute to differential reporting intensity across regions and limit the generalizability of the observed signal distribution to healthcare systems with distinct prescribing practices, regulatory frameworks, and medico‐legal contexts. Consequently, while the core associations identified in this analysis are likely robust, caution is warranted when extrapolating absolute signal strength or reporting frequency to non‐US populations.

Drug‐induced dystonia typically appears within days to weeks after treatment initiation, particularly with antipsychotics and antiemetic drugs such as metoclopramide [2, 44]. Our research indicates most adverse reactions (66.34%) occurred within 30 days following drug exposure. Among the drugs identified, metoclopramide, and risperidone showed the shortest median TTO (2 days), followed by aripiprazole (5 days). Notably, the estimated median onset time and 95% CI reflect variability in real‐world reporting. The 95% CI for olanzapine (11.33–22.92) and quetiapine (13.64–26.52) were distinctly separated from those of metoclopramide (1.52–2.70) and risperidone (1.41–2.90), indicating relatively stable intergroup differences, whereas the wider and overlapping interval observed for aripiprazole indicates greater uncertainty in its onset‐time estimate. These findings characterize the temporal patterns of this event across different drugs in real‐world settings.

Based on the event onset patterns observed in this study, we suggest that a 30‐day, risk‐stratified monitoring period may be considered for patients receiving medications associated with a higher reporting signal, with the goal of preventing drug‐induced AEs. Currently, there is a lack of systematic clinical guidelines for the management of drug‐induced dystonia. The following measures are proposed as potentially beneficial, as indicated by extant literature: baseline assessment of motor function; enhanced on‐site or telemedicine follow‐up during the initial 2 weeks of treatment; and the use of anticholinergic agents as prophylactic or rescue therapy in selected high‐risk patients [11, 45]. It should also be noted that the TTO analysis was based on reports with complete temporal information, representing only 20.49% of the total reports (5657/26,718). Consequently, the estimated onset‐time distribution may not fully reflect the underlying population pattern and is primarily applicable to cases with adequate documentation. This limitation underscores the need for more standardized and comprehensive electronic reporting systems for dystonia to improve the completeness and quality of postmarketing pharmacovigilance data.

In the drug‐drug interaction analysis, the combination of risperidone and aripiprazole was associated with the highest number of reported AEs for dystonia, and the greatest frequency of targeted AEs when co‐administered with other drugs. Although this combination has not been systematically evaluated with respect to dystonia risk, existing evidence suggests that both agents are primarily metabolized by CYP2D6. Competitive inhibition at this pathway may increase plasma concentrations of either or both drugs, thereby potentially heightening the risk of adverse neurological reactions, including dystonia [46]. However, the present dataset did not allow for a quantitative evaluation of this interaction. In addition, SSRIs have been reported to potentiate the effects of several antipsychotic drugs, including haloperidol, aripiprazole, risperidone, and quetiapine, partly through inhibition of hepatic CYP450 enzymes [46]. Preclinical studies further indicate that SSRIs may enhance 5‐HT2C receptor activity, leading to suppression of dopaminergic neurotransmission and a subsequent increase in dystonia susceptibility [47]. These findings highlight the need for careful risk assessment and enhanced surveillance when drugs are combined. In clinical practice, routine screening for potential drug–drug interactions, identification of high‐risk combinations, and pharmacist‐led medication review may help mitigate this risk. Nonetheless, the biological mechanisms underlying drug‐induced dystonia remain incompletely understood, and further mechanistic studies are warranted to clarify the pathways involved.

We also identified eight drugs whose labels did not indicate dystonia risk; these drugs included anti‐epileptics, antidepressants, anti‐Parkinson drugs, inhaled general anesthetics, and a new drug for treating the rare disease Rett syndrome. As previously outlined, dopaminergic agents may underlie the pathogenesis of dystonia, and sevoflurane may perturb the dopamine system. Other drugs, such as amantadine, may trigger rare adverse reactions in specific populations, including individuals with rare diseases or genetic predispositions. These findings broaden the range of drugs that can cause dystonia and require more vigilance from clinicians when prescribing. As an important medium to convey safety information, drug labels should comprehensively reflect potential AEs to guide clinical practice. Regulatory agencies should review safety data to validate risk signals and determine whether to include them on product labels to alert clinicians and patients. Notably, among the eight agents currently lacking dystonia warnings, four were supported by relatively few reports (30–39 cases each; Figure 1). Such limited evidence is insufficient to justify immediate label modification. Rather, these signals should prompt focused pharmacoepidemiologic or mechanistic studies to better define incidence, patient susceptibility, and causal relationships before regulatory action is considered.

Drug‐induced dystonia can often be mitigated by suspending the offending medication, decreasing the dosage, or switching to alternative medications with lower risk profiles. Early and accurate recognition is essential to prevent symptom persistence and long‐term functional impairment [48]. In this context, the use of validated assessment instruments, such as the Extrapyramidal Symptom Rating Scale Abbreviated Form (ESRS‐A), is recommended to support standardized clinical evaluation [49]. To prevent or mitigate drug‐induced dystonia, anticholinergic drugs, as well as neurotoxin injections and surgical interventions, can be used [1, 11]. Additionally, the combined use of multiple antipsychotic drugs should be minimized. If necessary, individual differences should be monitored, as appropriate, and the dose should be decreased to ensure the prevention of adverse reactions. Beyond pharmacological management, nonpharmacological rehabilitation strategies for focal dystonia have gained increasing attention. Interventions such as sensorimotor retraining, sensory discrimination training, constraint‐induced therapy, and movement control retraining have been reported to improve motor performance and alleviate dystonia symptoms [50]. Furthermore, accumulating evidence suggests that rehabilitation programs targeting neural plasticity may contribute to sustained improvements in motor function and associated movement impairments [51].

This study has several inherent limitations that should be carefully considered when interpreting the findings. First, FAERS is a spontaneous reporting system and is therefore subject to underreporting, incomplete information, reporting inconsistencies, and potential residual duplicate records, despite the application of standardized data‐cleaning and deduplication procedures. These factors may influence the absolute magnitude of disproportionality metrics. Second, reporting bias is an important concern in pharmacovigilance analyses. Differential reporting behaviors, including notoriety bias and litigation‐driven reporting, may lead to disproportionate reporting of certain drugs and artificially inflate signal strength. In the present study, lawyer‐submitted reports accounted for a substantial proportion of cases, which may have amplified the apparent RORs for specific agents. Although sensitivity analyses were conducted to assess the impact of this bias, its complete elimination is not possible, and the magnitude of individual drug signals may therefore be affected. Third, indication bias cannot be excluded, as dystonia‐related symptoms may be associated with the underlying disease for which the drug was prescribed rather than the drug exposure itself. This may confound signal interpretation and contribute to either overestimation or underestimation of drug–event associations. Fourth, the detected ADE signals were from only preliminary screening, and the ROR, PRR, MGPS, and BCPNN methods were combined to evaluate the association between drugs and dystonia, thus raising the screening threshold for target ADE signals; however, potential false positive signals cannot be ruled out [52].

Finally, although we identified signals between certain drugs and dystonia, these drugs do not necessarily directly cause dystonia. Spontaneous reporting systems do not capture the number of exposed individuals or person‐time; consequently, incidence rates cannot be calculated. Therefore, the observed ROR should be regarded as a hypothesis‐generating signal, and causality should be further evaluated in longitudinal cohort studies. Data mining of the FAERS database can reveal new associations and confirm known associations between drugs and adverse reactions, and aid in identifying potentially risky medications. These insights may aid in clinical decision‐making in the absence of more extensive research.

## 5 Conclusion

Metoclopramide was the most frequently implicated drug among the top 50 drugs associated with dystonia, and had the highest ROR signal, according to the FAERS database. However, this finding should be interpreted with caution, as the apparent elevation in risk is likely influenced, at least in part, by litigation‐driven reporting bias. Antipsychotics, including aripiprazole, risperidone, quetiapine, olanzapine, and haloperidol, also demonstrated significant associations with dystonia, thus highlighting the need for caution when using these drugs, particularly in combination therapies. Although this study did not estimate the incidence of dystonia, the results emphasize the importance of judicious prescribing and close clinical monitoring in patients at increased risk of drug‐induced dystonia. Healthcare providers should consider adjusting medications or dosages for at‐risk patients to minimize the likelihood of dystonia onset or exacerbation. Additionally, this study highlights gaps in drug labeling: several implicated drugs did not list dystonia as a known risk, thus underscoring a need to optimize drug safety communication to both clinicians and the public. Future research should quantify the incidence of dystonia caused by metoclopramide, antipsychotic drugs (e.g., aripiprazole), and other drug classes (e.g., antiepileptic drugs) in large‐scale clinical trials or retrospective cohort studies, and should construct risk prediction models incorporating patient characteristics (e.g., age and sex) to facilitate accurate clinical decision‐making.

## Author Contributions

**Chunhua Chen:** conceptualization, methodology, writing – original draft, software, formal analysis, investigation, data curation. **Xiaobin Lin:** conceptualization, writing – review and editing, data curation, formal analysis. **Yi Yang:** writing – review and editing, resources. **Jiajia Yan:** writing – review and editing. **Jingxiu Chen:** writing – review and editing. **Yifan Zheng:** writing—review and editing, supervision. **Jia Li:** conceptualization, design, funding acquisition, writing—review and editing, supervision, formal analysis. All authors have read and approved the final version of the manuscript. Jia Li had full access to all of the data in this study and takes complete responsibility for the integrity of the data and the accuracy of the data analysis.

## Ethics Statement

The FAERS Public Dashboard is an accessible online platform that aggregates mandatory data submissions from pharmaceutical companies and voluntary adverse drug reaction (ADR) reports from consumers and healthcare professionals. As these data are publicly available and allow unrestricted reuse through an open licence, this study did not require permission from the database/repository owner. In addition, this study maintained the anonymity of the data and therefore did not require obtaining written informed consent from participants. Thus, the analysis does not require informed consent or ethical approval by an ethical standards committee for human experimentation.

## Conflicts of Interest

The authors declare no conflicts of interest.

## Transparency Statement

The lead author Yifan Zheng, Jia Li affirm that this manuscript is an honest, accurate, and transparent account of the study being reported; that no important aspects of the study have been omitted; and that any discrepancies from the study as planned (and, if relevant, registered) have been explained.

## Supporting information


**Supplementary Figure 1:** Number of reported cases of dystonia from the first quarter of 2004 to the third quarter of 2024. **Supplementary Table 1:** PTs and their MedDRA codes obtained by SMQ_narrow and SMQ_broad query for dystonia. **Supplementary Table 2:** Top 25 indications for patients in the report. **Supplementary Table 3:** Signal strength of positive signals associated with dystonia drug grade ADE (*n*=96). **Supplementary Table 4:** Signal strength of ADEs at the Anatomical Therapeutic Chemical Classification System (ATC) level in the FAERS database. **Supplementary Table 5:** Key Characteristics of drug‐induced dystonia: A comparative analysis based on lawyer reports and exclusion of lawyer reports. **Supplementary Table 6:** Signal strength of positive signals associated with dystonia drug grade ADE after excluding lawyer's reports (*n*=111).

## Data Availability

The raw data underlying this study are publicly available in the FAERS repository https://fis.fda.gov/extensions/FPD-QDE-FAERS/FPD-QDE-FAERS.html. The data extraction, processing, and cleaning scripts used to generate the analyzed datasets will be provided by the corresponding author upon reasonable request.
