# Supplementary material for: Identifying High‐Risk Medications for Drug‐Induced Dystonia: A 20‐Year Retrospective Real‐World Pharmacovigilance Study Based on FAERS
Source: Health Sci Rep. 2026 Apr 15;9(4):e72194. doi: 10.1002/hsr2.72194 (PMC13083585; doi:10.1002/hsr2.72194)
Supplement: Supplementary file 1 — Supplementary Figure 1: Number of reported cases of dystonia from the first quarter of 2004 to the third quarter of 2024. Supplementary Table 1: PTs and their MedDRA codes obtained by SMQ_narrow and SMQ_broad query for dystonia. Supplementary Table 2: Top 25 indications for patients in the report. Supplementary Table 3: Signal strength of positive signals associated with dystonia drug grade ADE (n=96). Supplementary Table 4: Signal strength of ADEs at the Anatomical Therapeutic Chemical Classification System (ATC) level in the FAERS database. Supplementary Table 5: Key Characteristics of drug‐induced dystonia: A comparative analysis based on lawyer reports and exclusion of lawyer reports. Supplementary Table 6: Signal strength of positive signals associated with dystonia drug grade ADE after excluding lawyer's reports (n=111). [file HSR2-9-e72194-s001.docx]

Supplementary materials

**Identifying high-risk medications for drug-induced dystonia: A 20-year retrospective pharmacovigilance study**

**Supplementary Table 1** PTs and their MedDRA codes obtained by SMQ_ narrow and SMQ_broad query for dystonia

**Supplementary Table 2** Top 25 indications for patients in the report

**Supplementary Table 3** Signal strength of positive signals associated with dystonia drug grade ADE (*n*=96)

**Supplementary Table 4** Signal strength of ADEs at the Anatomical Therapeutic Chemical Classification System (ATC) level in FAERS database

**Supplementary Table 5** Key Characteristics of drug-induced dystonia: A comparative analysis based on lawyer reports and exclusion of lawyer reports

**Supplementary Table 6** Signal strength of positive signals associated with dystonia drug grade ADE after excluding lawyer's reports (*n*=111)

**Supplementary Fig.1.** Number of reported cases of dystonia from the first quarter of 2004 to the third quarter of 2024

**Supplementary Table 1** PTs and their MedDRA codes obtained by SMQ_narrow and SMQ_broad query for dystonia

| Search method | PT name（MedDRA code） |
| --- | --- |
| SMQ_broad (*n*=42) | Meige's syndrome (10027138)、Pleurothotonus (10035628)、Dystonia (10013983)、Status dystonicus (10088053)、Dystonic tremor(10073210)、Opisthotonus(10030899)、Spasmodic dysphonia(10067672)、Emprosthotonus(10014566)、Writer's cramp(10072249)、Oromandibular dystonia(10067954)、Pharyngeal dystonia(10081226)、Torticollis(10044074)、Trismus(10044684)、Oculogyric crisis(10030071)  、Blepharospasm (10005159)、Chronic tic disorder (10076661)、Complex tic (10076663)、Drooling (10013642)、Extrapyramidal disorder (10015832)、Facial spasm (10063006)、Gait inability (10017581)、Laryngospasm (10023891)、Motor dysfunction (10061296)、Movement disorder (10028035)、Muscle contractions involuntary (10028293)、Muscle spasms (10028334)、Muscle spasticity (10028335)、Muscle tightness (10049816)、Muscle tone disorder (10072889)、Muscle twitching (10028347)、Musculoskeletal stiffness (10052904)、Oesophageal spasm (10030184)、Oropharyngeal spasm (10031111)、Posture abnormal (10036436)、Posturing (10036437)、Provisional tic disorder (10076694)、Risus sardonicus (10039198)、Secondary tic (10076702)、Tic (10043833)、Tongue spasm (10043981)、Torticollis psychogenic (10044076)、Uvular spasm (10050908) |
| SMQ_narrow (*n*=14) | Meige's syndrome (10027138)、Pleurothotonus (10035628)、Dystonia (10013983)、Status dystonicus (10088053)、Dystonic tremor(10073210)、Opisthotonus(10030899)、Spasmodic dysphonia(10067672)、Emprosthotonus(10014566)、Writer's cramp(10072249)、Oromandibular dystonia(10067954)、Pharyngeal dystonia(10081226)、Torticollis(10044074)、Trismus(10044684)、Oculogyric crisis(10030071) |

**Supplementary Table 2** Top 25 indications for patients in the report

| **Indication** | **Reports, *n* (%)** |
| --- | --- |
| Abdominal distension/Dyspepsia | 1,930(10.42%) |
| Schizophrenia | 1,526(8.24%) |
| Abdominal distension | 1,436(7.76%) |
| Parkinson's disease | 1,057(5.71%) |
| Depression | 773(4.18%) |
| Bipolar disorder | 643(3.47%) |
| Psychotic disorder | 394(2.13%) |
| Attention deficit hyperactivity disorder | 378(2.04%) |
| Multiple sclerosis | 276(1.49%) |
| Osteoporosis | 241(1.30%) |
| Anxiety | 229(1.24%) |
| Nausea | 217(1.17%) |
| Schizoaffective disorder | 217(1.17%) |
| Major depression | 189(1.02%) |
| Rheumatoid arthritis | 186(1.00%) |
| Epilepsy | 174(0.94%) |
| Dementia Alzheimer's type | 163(0.88%) |
| Migraine | 134(0.72%) |
| Agitation | 126(0.68%) |
| Bipolar I disorder | 126(0.68%) |
| Gastrooesophageal reflux disease | 125(0.68%) |
| Muscle spasticity | 123(0.66%) |
| Hypertension | 109(0.59%) |
| Tardive dyskinesia | 100(0.54%) |
| Crohn's disease | 99(0.53%) |

**Supplementary Table 3** Signal strength of positive signals associated with dystonia drug grade ADE (*n*=96)

| Drug NAME | ATC Code(I) | Case reports | ROR (95% CI) | PRR (95% CI) | Chi-square | IC(IC_025)_ | EBGM(EBGM_05_) |
| --- | --- | --- | --- | --- | --- | --- | --- |
| metoclopramide | A | 7,178 | 155.90(151.70,160.21) | 146.77(142.99,150.65) | 781861 | 6.79(6.73) | 110.61(107.63) |
| aripiprazole | N | 1,595 | 14.73(14.00,15.49) | 14.62(13.91,15.38) | 19139.1 | 3.79(3.71) | 13.87(13.19) |
| risperidone | N | 1,366 | 11.85(11.22,12.51) | 11.78(11.16,12.44) | 12848.4 | 3.49(3.40) | 11.27(10.67) |
| quetiapine | N | 940 | 6.12(5.73,6.53) | 6.10(5.72,6.51) | 3883.06 | 2.57(2.47) | 5.94(5.56) |
| olanzapine | N | 837 | 8.55(7.98,9.16) | 8.52(7.95,9.12) | 5396.50 | 3.05(2.94) | 8.30(7.75) |
| haloperidol | N | 756 | 37.26(34.64,40.08) | 36.57(34.04,39.28) | 25485.3 | 5.16(4.98) | 35.64(33.13) |
| Carbidopa/levodopa | N | 755 | 6.75(6.28,7.26) | 6.73(6.26,7.24) | 3591.79 | 2.72(2.60) | 6.58(6.12) |
| sertraline | N | 514 | 5.27(4.83,5.75) | 5.26(4.82,5.74) | 1742.19 | 2.37(2.23) | 5.18(4.75) |
| paliperidone | N | 502 | 11.44(10.47,12.50) | 11.38(10.42,12.43) | 4672.36 | 3.49(3.33) | 11.20(10.25) |
| ziprasidone | N | 479 | 28.99(26.47,31.75) | 28.57(26.12,31.25) | 12541.3 | 4.81(4.60) | 28.12(25.67) |
| clozapine | N | 448 | 3.27(2.98,3.59) | 3.26(2.97,3.58) | 692.84 | 1.69(1.55) | 3.23(2.94) |
| ondansetron | A | 399 | 10.65(9.65,11.76) | 10.60(9.60,11.70) | 3422.18 | 3.39(3.21) | 10.47(9.48) |
| methylphenidate | N | 322 | 5.00(4.48,5.59) | 4.99(4.48,5.57) | 1017.72 | 2.31(2.13) | 4.95(4.43) |
| lurasidone | N | 283 | 13.08(11.63,14.71) | 13.00(11.57,14.61) | 3105.45 | 3.69(3.46) | 12.88(11.45) |
| lamotrigine | N | 253 | 2.49(2.20,2.82) | 2.49(2.20,2.81) | 222.92 | 1.31(1.12) | 2.47(2.19) |
| baclofen | N | 252 | 4.47(3.95,5.07) | 4.47(3.95,5.05) | 672.29 | 2.15(1.95) | 4.44(3.92) |
| fluoxetine | N | 247 | 4.83(4.26,5.47) | 4.82(4.25,5.46) | 741.13 | 2.26(2.05) | 4.78(4.22) |
| valproic acid | N | 225 | 2.93(2.57,3.34) | 2.93(2.57,3.33) | 282.99 | 1.54(1.34) | 2.91(2.55) |
| donepezil | N | 189 | 13.80(11.96,15.93) | 13.71(11.89,15.81) | 2213.59 | 3.77(3.46) | 13.63(11.80) |
| citalopram | N | 182 | 3.53(3.05,4.09) | 3.53(3.05,4.08) | 327.75 | 1.81(1.58) | 3.51(3.04) |
| escitalopram | N | 179 | 3.94(3.40,4.56) | 3.93(3.39,4.55) | 389.07 | 1.97(1.73) | 3.91(3.38) |
| pramipexole | N | 163 | 9.91(8.49,11.56) | 9.86(8.46,11.50) | 1291.04 | 3.29(2.99) | 9.81(8.41) |
| brexpiprazole | N | 155 | 12.19(10.41,14.29) | 12.12(10.36,14.19) | 1574.07 | 3.59(3.26) | 12.06(10.30) |
| asenapine | N | 153 | 16.97(14.47,19.91) | 16.83(14.37,19.71) | 2266.84 | 4.07(3.69) | 16.74(14.27) |
| mirtazapine | N | 121 | 3.36(2.81,4.02) | 3.36(2.81,4.01) | 199.54 | 1.74(1.45) | 3.35(2.80) |
| cetirizine | R | 119 | 2.64(2.20,3.16) | 2.64(2.20,3.16) | 120.44 | 1.39(1.11) | 2.63(2.20) |
| valbenazine | N | 110 | 4.94(4.10,5.96) | 4.93(4.09,5.94) | 343.40 | 2.30(1.97) | 4.91(4.07) |
| clonazepam | N | 108 | 2.44(2.02,2.94) | 2.43(2.02,2.94) | 91.00 | 1.28(0.98) | 2.43(2.01) |
| tetrabenazine | N | 91 | 9.46(7.69,11.62) | 9.41(7.67,11.56) | 682.45 | 3.23(2.80) | 9.39(7.64) |
| diazepam | N | 81 | 2.72(2.19,3.38) | 2.72(2.19,3.38) | 87.78 | 1.44(1.09) | 2.71(2.18) |
| propofol | N | 80 | 6.79(5.45,8.45) | 6.76(5.43,8.42) | 392.13 | 2.75(2.33) | 6.75(5.42) |
| prochlorperazine | N | 79 | 76.75(61.27,96.14) | 73.78(59.42,91.61) | 5659.16 | 6.20(4.94) | 73.58(58.74) |
| ropinirole | N | 79 | 5.99(4.80,7.47) | 5.97(4.79,7.45) | 326.40 | 2.58(2.16) | 5.96(4.78) |
| cariprazine | N | 78 | 13.97(11.18,17.47) | 13.88(11.12,17.32) | 930.18 | 3.79(3.25) | 13.84(11.08) |
| lorazepam | N | 76 | 2.51(2.01,3.15) | 2.51(2.00,3.14) | 68.87 | 1.33(0.97) | 2.51(2.00) |
| rivastigmine | N | 71 | 2.63(2.08,3.31) | 2.62(2.08,3.31) | 71.18 | 1.39(1.02) | 2.62(2.07) |
| promethazine | A | 63 | 9.60(7.50,12.31) | 9.56(7.47,12.23) | 482.13 | 3.25(2.71) | 9.54(7.45) |
| rotigotine | N | 61 | 4.80(3.73,6.17) | 4.79(3.73,6.15) | 182.53 | 2.26(1.80) | 4.78(3.72) |
| lithium | A | 58 | 3.74(2.89,4.83) | 3.73(2.88,4.83) | 115.74 | 1.90(1.46) | 3.72(2.88) |
| apomorphine | N | 45 | 3.68(2.75,4.93) | 3.68(2.75,4.92) | 87.61 | 1.88(1.37) | 3.67(2.74) |
| deutetrabenazine | N | 39 | 6.95(5.07,9.52) | 6.93(5.06,9.48) | 197.59 | 2.79(2.13) | 6.92(5.05) |
| carbidopa/entacapone/levodopa | N | 38 | 7.96(5.78,10.94) | 7.93(5.77,10.89) | 229.83 | 2.99(2.29) | 7.92(5.76) |
| trofinetide | N | 36 | 5.26(3.79,7.30) | 5.25(3.79,7.28) | 123.86 | 2.39(1.76) | 5.25(3.78) |
| amitriptyline | N | 34 | 3.47(2.47,4.85) | 3.46(2.47,4.84) | 59.44 | 1.79(1.20) | 3.46(2.47) |
| amantadine | N | 32 | 6.06(4.29,8.58) | 6.05(4.28,8.55) | 134.75 | 2.60(1.89) | 6.04(4.27) |
| galantamine | N | 31 | 6.99(4.91,9.95) | 6.97(4.90,9.91) | 158.46 | 2.80(2.04) | 6.96(4.89) |
| sevoflurane | N | 30 | 6.10(4.26,8.73) | 6.08(4.25,8.69) | 127.27 | 2.60(1.86) | 6.07(4.24) |
| entacapone | N | 29 | 7.46(5.18,10.75) | 7.44(5.17,10.70) | 161.56 | 2.89(2.09) | 7.43(5.16) |
| opicapone | N | 28 | 26.19(18.04,38.04) | 25.85(17.89,37.35) | 668.51 | 4.69(3.26) | 25.82(17.78) |
| articaine/epinephrine | N | 26 | 12.18(8.28,17.92) | 12.11(8.25,17.77) | 264.89 | 3.60(2.54) | 12.10(8.23) |
| midazolam | N | 25 | 5.27(3.56,7.81) | 5.26(3.56,7.78) | 86.25 | 2.39(1.61) | 5.26(3.55) |
| chlorpromazine | N | 24 | 16.84(11.27,25.18) | 16.70(11.21,24.88) | 354.22 | 4.06(2.78) | 16.69(11.17) |
| lumateperone | N | 24 | 5.30(3.55,7.91) | 5.29(3.54,7.88) | 83.39 | 2.40(1.59) | 5.28(3.54) |
| Cilastatin/imipenem | N | 23 | 4.17(2.77,6.27) | 4.16(2.76,6.26) | 55.18 | 2.06(1.29) | 4.16(2.76) |
| rasagiline | J | 23 | 5.87(3.90,8.85) | 5.86(3.90,8.81) | 92.66 | 2.55(1.69) | 5.86(3.89) |
| fluphenazine | N | 21 | 23.96(15.58,36.85) | 23.67(15.47,36.21) | 455.88 | 4.56(2.92) | 23.65(15.38) |
| cabergoline | N | 19 | 3.65(2.33,5.73) | 3.65(2.33,5.72) | 36.51 | 1.87(1.04) | 3.65(2.32) |
| droperidol | N | 19 | 57.83(36.63,91.30) | 56.13(36.04,87.42) | 1028.70 | 5.81(3.25) | 56.09(35.53) |
| eletriptan | N | 19 | 4.07(2.59,6.38) | 4.06(2.59,6.36) | 43.82 | 2.02(1.17) | 4.06(2.59) |
| clomipramine | M | 18 | 6.65(4.18,10.56) | 6.63(4.18,10.51) | 85.97 | 2.73(1.69) | 6.62(4.17) |
| penicillamine | N | 18 | 22.56(14.17,35.92) | 22.31(14.09,35.32) | 366.32 | 4.48(2.73) | 22.29(14.01) |
| bromocriptine | R | 15 | 5.27(3.17,8.75) | 5.26(3.17,8.71) | 51.69 | 2.39(1.33) | 5.25(3.16) |
| fluvoxamine | A | 15 | 5.10(3.07,8.46) | 5.09(3.07,8.43) | 49.26 | 2.35(1.30) | 5.08(3.06) |
| ketamine | N | 15 | 4.49(2.70,7.45) | 4.48(2.70,7.42) | 40.52 | 2.16(1.16) | 4.48(2.70) |
| thiethylperazine | N | 15 | 220.04(128.87,375.72) | 197.06(122.07,318.13) | 2926.07 | 7.62(3.13) | 196.96(115.35) |
| buspirone | N | 14 | 4.40(2.60,7.44) | 4.39(2.60,7.41) | 36.69 | 2.13(1.09) | 4.39(2.60) |
| iloperidone | N | 14 | 11.65(6.89,19.70) | 11.58(6.87,19.52) | 135.34 | 3.53(2.02) | 11.58(6.84) |
| miglustat | A | 14 | 6.03(3.57,10.18) | 6.01(3.56,10.14) | 58.47 | 2.59(1.42) | 6.01(3.55) |
| perphenazine | N | 14 | 29.14(17.19,49.41) | 28.71(17.07,48.29) | 374.47 | 4.84(2.58) | 28.70(16.93) |
| cerliponase alfa | A | 13 | 22.85(13.22,39.49) | 22.59(13.16,38.78) | 268.28 | 4.50(2.37) | 22.58(13.07) |
| imipramine | N | 12 | 6.21(3.53,10.95) | 6.20(3.52,10.90) | 52.31 | 2.63(1.34) | 6.20(3.51) |
| pimozide | N | 11 | 31.72(17.48,57.57) | 31.21(17.37,56.09) | 321.73 | 4.96(2.31) | 31.20(17.19) |
| viloxazine | N | 9 | 10.73(5.57,20.66) | 10.67(5.56,20.47) | 78.91 | 3.42(1.52) | 10.67(5.54) |
| Bupropion/dextromethorphan | N | 8 | 4.78(2.39,9.56) | 4.77(2.39,9.53) | 23.83 | 2.25(0.79) | 4.77(2.38) |
| palonosetron | A | 8 | 9.04(4.51,18.11) | 9.00(4.51,17.97) | 56.92 | 3.17(1.29) | 9.00(4.49) |
| tiotixene | N | 8 | 18.40(9.17,36.93) | 18.23(9.15,36.34) | 130.35 | 4.19(1.68) | 18.23(9.09) |
| amisulpride | N | 7 | 27.60(13.09,58.23) | 27.22(13.05,56.79) | 176.84 | 4.77(1.64) | 27.21(12.90) |
| benzatropine | N | 7 | 9.99(4.75,21.00) | 9.94(4.75,20.82) | 56.33 | 3.31(1.21) | 9.94(4.73) |
| etomidate | N | 7 | 17.31(8.22,36.44) | 17.16(8.21,35.88) | 106.58 | 4.10(1.48) | 17.16(8.15) |
| pergolide | N | 7 | 5.91(2.81,12.40) | 5.89(2.81,12.34) | 28.43 | 2.56(0.85) | 5.89(2.80) |
| procyclidine | M | 7 | 52.14(24.60,110.51) | 50.76(24.44,105.42) | 341.56 | 5.67(1.78) | 50.75(23.95) |
| suxamethonium | M | 7 | 5.95(2.83,12.50) | 5.94(2.83,12.44) | 28.73 | 2.57(0.85) | 5.93(2.83) |
| vecuronium | N | 7 | 9.75(4.64,20.49) | 9.70(4.63,20.31) | 54.65 | 3.28(1.19) | 9.70(4.62) |
| methylergometrine | G | 6 | 4.94(2.22,11.02) | 4.93(2.22,10.97) | 18.82 | 2.30(0.57) | 4.93(2.21) |
| trihexyphenidyl | N | 5 | 13.29(5.52,32.04) | 13.21(5.51,31.63) | 56.43 | 3.72(0.94) | 13.20(5.48) |
| dolasetron | A | 4 | 39.51(14.68,106.38) | 38.72(14.68,102.13) | 147.04 | 5.27(0.87) | 38.72(14.38) |
| frovatriptan | N | 4 | 11.17(4.18,29.86) | 11.11(4.18,29.52) | 36.82 | 3.47(0.58) | 11.11(4.16) |
| loxapine | N | 4 | 27.10(10.10,72.73) | 26.73(10.10,70.74) | 99.12 | 4.74(0.82) | 26.73(9.96) |
| safinamide | N | 4 | 10.00(3.74,26.71) | 9.95(3.74,26.44) | 32.21 | 3.31(0.54) | 9.95(3.72) |
| thioridazine | N | 4 | 8.45(3.16,22.55) | 8.41(3.16,22.37) | 26.13 | 3.07(0.47) | 8.41(3.15) |
| Dexmethyl phenidate/serdexmethylphenidate | N | 3 | 6.56(2.11,20.39) | 6.54(2.11,20.25) | 14.09 | 2.71(0.01) | 6.54(2.11) |
| domperidone | A | 3 | 11.90(3.83,37.04) | 11.84(3.83,36.57) | 29.77 | 3.56(0.22) | 11.83(3.80) |
| molindone | A | 3 | 92.31(28.96,294.20) | 88.03(29.16,265.72) | 258.22 | 6.46(0.46) | 88.02(27.62) |
| nabilone | A | 3 | 10.18(3.27,31.67) | 10.13(3.28,31.32) | 24.70 | 3.34(0.18) | 10.13(3.26) |
| pentazocine | N | 3 | 7.80(2.51,24.24) | 7.77(2.51,24.04) | 17.71 | 2.96(0.08) | 7.77(2.50) |
| trientine | N | 3 | 8.61(2.77,26.77) | 8.57(2.77,26.52) | 20.08 | 3.10(0.12) | 8.57(2.76) |

*Note1: ranked by case reports*

*Note2: Signals are detected when all the following criteria are met: a ≥ 3, PRR ≥2 and Chi-Square ≥ 4, lower limit of 95% CI of ROR > 1, IC_025_ > 0, EBGM_05_ > 2*

**Supplementary Table 4** Signal strength of ADEs at the Anatomical Therapeutic Chemical Classification System (ATC) level in the FAERS database

| ATC NAME(I) | ATC Code(I) | Case reports | ROR (95% CI) | PRR (95% CI) | Chi-Square | IC (IC_025_) | EBGM(EBGM_05_) |
| --- | --- | --- | --- | --- | --- | --- | --- |
| nervous system | N | 16,036 | 5.33(5.21,5.45) | 5.32(5.20,5.45) | 25100.6 | 1.55(1.52) | 2.93(2.86) |
| alimentary tract and metabolism | A | 8,877 | 2.87(2.80,2.94) | 2.87(2.79,2.94) | 7478.31 | 1.20(1.16) | 2.29(2.24) |
| antineoplastic and immunomodulating agents | L | 1,929 | 0.12(0.12,0.13) | 0.12(0.12,0.13) | 11240.8 | -2.46(-2.53) | 0.18(0.17) |
| musculo-skeletal system | M | 1,403 | 0.88(0.83,0.93) | 0.88(0.83,0.93) | 22.47 | -0.18(-0.26) | 0.88(0.84) |
| dermatologicals | D | 1,305 | 0.39(0.37,0.41) | 0.39(0.37,0.41) | 1178.24 | -1.25(-1.34) | 0.42(0.40) |
| genito urinary system and sex hormones | G | 1,301 | 0.52(0.49,0.55) | 0.52(0.49,0.55) | 547.95 | -0.88(-0.96) | 0.54(0.51) |
| sensory organs | S | 793 | 0.39(0.36,0.41) | 0.39(0.36,0.41) | 757.17 | -1.31(-1.42) | 0.40(0.37) |
| respiratory system | R | 718 | 0.32(0.30,0.34) | 0.32(0.30,0.34) | 1014.03 | -1.57(-1.68) | 0.34(0.31) |
| antiinfectives for systemic use | J | 700 | 0.39(0.36,0.42) | 0.39(0.36,0.42) | 653.04 | -1.31(-1.41) | 0.40(0.38) |
| cardiovascular system | C | 676 | 0.23(0.21,0.25) | 0.23(0.21,0.25) | 1733.70 | -2.03(-2.14) | 0.25(0.23) |
| various | V | 296 | 0.35(0.32,0.40) | 0.35(0.32,0.40) | 343.61 | -1.47(-1.63) | 0.36(0.32) |
| systemic hormonal preparations, excl. sex hormones and insulins | H | 253 | 0.22(0.20,0.25) | 0.22(0.20,0.25) | 671.74 | -2.11(-2.29) | 0.23(0.20) |
| blood and blood forming organs | B | 249 | 0.14(0.12,0.16) | 0.14(0.12,0.16) | 1331.11 | -2.78(-2.96) | 0.15(0.13) |
| antiparasitic products, insecticides and repellents | P | 70 | 0.66(0.52,0.83) | 0.66(0.52,0.83) | 12.53 | -0.60(-0.94) | 0.66(0.52) |

*Note: ranked by case reports*

**Supplementary Table 5** Key Characteristics of drug-induced dystonia: A comparative analysis based on lawyer reports and exclusion of lawyer reports

| **Characteristics** | **Number of cases, *n* (%)** | |
| --- | --- | --- |
|  | Reported by Lawyers  6,869(24.87) | Excluding Lawyer's Reports  20,749 (75.13) |
| **Sex** |  |  |
| Female | 4,237 (61.68) | 10,909 (52.58) |
| Male | 2,004 (29.17) | 7,932 (38.23) |
| Not specified | 628 (9.14) | 1,908 (9.20) |
| **Age (years)** |  |  |
| <18 | 32 (0.47) | 2,875 (13.86) |
| 18–44 | 22 (0.32) | 5,807 (27.99) |
| 45–64 | 46 (0.67) | 3,546 (17.09) |
| ≥65 | 16 (0.23) | 2,852 (13.75) |
| Unknown | 6,753 (98.31) | 5,669 (27.32) |
| **Top five reporting countries** |  |  |
| United States of America | 6,829 (99.42) | 8,671 (41.79) |
| United Kingdom | 19 (0.28) | 2,164 (10.53) |
| Canada | 5 (0.07) | 1,271 (6.13) |
| Italy | 4 (0.06) | 772 (3.72) |
| Germany | 2 (0.03) | 766 (3.69) |

**Supplementary Table 6** Signal strength of positive signals associated with dystonia drug grade ADE after excluding lawyer's reports (*n*=111)

| Drug Name | ATC Code(I) | Case reports | ROR (95% CI) | PRR (95% CI) | Chi-square | IC(IC_025)_ | EBGM(EBGM_05_) |
| --- | --- | --- | --- | --- | --- | --- | --- |
| aripiprazole | N | 1,587 | 19.59 (18.61, 20.62) | 19.45 (18.48, 20.46) | 2421.14 | 4.18 (4.09) | 18.12 (17.22) |
| risperidone | N | 1,340 | 15.48 (14.64, 16.36) | 15.39 (14.57, 16.26) | 1512.84 | 3.86 (3.76) | 14.52 (13.74) |
| quetiapine | N | 936 | 8.08 (7.57, 8.63) | 8.06 (7.55, 8.61) | 385.1 | 2.96 (2.85) | 7.76 (7.27) |
| olanzapine | N | 835 | 11.3 (10.55, 12.12) | 11.26 (10.51, 12.06) | 789.73 | 3.44 (3.32) | 10.87 (10.14) |
| Carbidopa/levodopa | N | 755 | 8.94 (8.31, 9.62) | 8.91 (8.29, 9.58) | 478.98 | 3.11 (2.99) | 8.64 (8.04) |
| haloperidol | N | 752 | 49.06 (45.59, 52.79) | 48.15 (44.8, 51.75) | 12250.65 | 5.54 (5.35) | 46.54 (43.25) |
| metoclopramide | A | 532 | 11.69 (10.72, 12.74) | 11.64 (10.68, 12.68) | 817.54 | 3.51 (3.35) | 11.38 (10.44) |
| sertraline | N | 503 | 6.81 (6.23, 7.44) | 6.79 (6.22, 7.42) | 258.26 | 2.74 (2.59) | 6.66 (6.09) |
| paliperidone | N | 502 | 15.1 (13.82, 16.51) | 15.02 (13.75, 16.41) | 1345.55 | 3.88 (3.71) | 14.7 (13.45) |
| ziprasidone | N | 477 | 38.1 (34.77, 41.75) | 37.55 (34.31, 41.09) | 6897.78 | 5.2 (4.96) | 36.76 (33.55) |
| clozapine | N | 448 | 4.31 (3.93, 4.73) | 4.31 (3.92, 4.73) | 87.18 | 2.08 (1.94) | 4.24 (3.86) |
| ondansetron | A | 398 | 14.01 (12.68, 15.47) | 13.94 (12.63, 15.39) | 1114.81 | 3.78 (3.59) | 13.7 (12.41) |
| methylphenidate | N | 321 | 6.57 (5.89, 7.34) | 6.56 (5.87, 7.32) | 228.91 | 2.7 (2.51) | 6.48 (5.8) |
| lurasidone | N | 282 | 17.17 (15.26, 19.31) | 17.06 (15.17, 19.17) | 1479.11 | 4.07 (3.82) | 16.85 (14.98) |
| lamotrigine | N | 253 | 3.28 (2.89, 3.71) | 3.27 (2.89, 3.7) | 40.74 | 1.7 (1.5) | 3.25 (2.87) |
| baclofen | N | 252 | 5.89 (5.2, 6.67) | 5.88 (5.19, 6.65) | 173.46 | 2.54 (2.33) | 5.82 (5.14) |
| fluoxetine | N | 244 | 6.28 (5.53, 7.12) | 6.26 (5.52, 7.1) | 198.6 | 2.63 (2.42) | 6.2 (5.47) |
| valproic acid | N | 223 | 3.82 (3.35, 4.36) | 3.81 (3.34, 4.35) | 60.61 | 1.92 (1.71) | 3.79 (3.32) |
| donepezil | N | 189 | 18.16 (15.73, 20.96) | 18.03 (15.64, 20.8) | 1393.5 | 4.16 (3.83) | 17.89 (15.49) |
| citalopram | N | 182 | 4.65 (4.02, 5.38) | 4.64 (4.01, 5.37) | 95.52 | 2.2 (1.96) | 4.61 (3.98) |
| escitalopram | N | 178 | 5.15 (4.44, 5.97) | 5.14 (4.44, 5.96) | 120.76 | 2.35 (2.1) | 5.11 (4.41) |
| pramipexole | N | 163 | 13.03 (11.16, 15.21) | 12.97 (11.12, 15.12) | 744.53 | 3.69 (3.36) | 12.88 (11.03) |
| brexpiprazole | N | 155 | 16.04 (13.69, 18.79) | 15.94 (13.62, 18.66) | 1036.22 | 3.98 (3.62) | 15.83 (13.51) |
| asenapine | N | 153 | 22.32 (19.02, 26.18) | 22.13 (18.89, 25.92) | 1737.56 | 4.46 (4.04) | 21.98 (18.73) |
| cetirizine | R | 119 | 3.47 (2.9, 4.15) | 3.47 (2.89, 4.15) | 42.87 | 1.79 (1.49) | 3.45 (2.88) |
| mirtazapine | N | 118 | 4.31 (3.6, 5.16) | 4.3 (3.59, 5.16) | 73.18 | 2.1 (1.79) | 4.29 (3.58) |
| valbenazine | N | 110 | 6.49 (5.38, 7.83) | 6.48 (5.37, 7.81) | 175.11 | 2.69 (2.35) | 6.45 (5.35) |
| clonazepam | N | 108 | 3.2 (2.65, 3.87) | 3.2 (2.65, 3.86) | 34.03 | 1.67 (1.37) | 3.19 (2.64) |
| tetrabenazine | N | 91 | 12.43 (10.11, 15.27) | 12.37 (10.07, 15.19) | 521.2 | 3.62 (3.15) | 12.32 (10.02) |
| diazepam | N | 81 | 3.58 (2.87, 4.45) | 3.57 (2.87, 4.44) | 42.28 | 1.83 (1.47) | 3.56 (2.86) |
| propofol | N | 80 | 8.92 (7.16, 11.11) | 8.89 (7.14, 11.07) | 278.75 | 3.15 (2.69) | 8.86 (7.11) |
| prochlorperazine | N | 79 | 100.84 (80.5, 126.34) | 96.93 (78.06, 120.37) | 6895.38 | 6.59 (5.13) | 96.59 (77.1) |
| ropinirole | N | 79 | 7.87 (6.31, 9.82) | 7.85 (6.29, 9.78) | 221.74 | 2.97 (2.53) | 7.82 (6.27) |
| cariprazine | N | 78 | 18.36 (14.69, 22.95) | 18.23 (14.61, 22.76) | 859.94 | 4.18 (3.57) | 18.17 (14.54) |
| lorazepam | N | 76 | 3.3 (2.63, 4.13) | 3.3 (2.63, 4.13) | 33.9 | 1.72 (1.35) | 3.29 (2.63) |
| rivastigmine | N | 71 | 3.45 (2.73, 4.36) | 3.45 (2.73, 4.35) | 37.22 | 1.78 (1.39) | 3.44 (2.72) |
| promethazine | A | 63 | 12.62 (9.85, 16.17) | 12.56 (9.81, 16.07) | 429.49 | 3.65 (3.05) | 12.53 (9.78) |
| rotigotine | N | 61 | 6.3 (4.9, 8.11) | 6.29 (4.89, 8.08) | 130.06 | 2.65 (2.16) | 6.27 (4.88) |
| lithium | A | 58 | 4.91 (3.79, 6.35) | 4.9 (3.79, 6.34) | 77.39 | 2.29 (1.82) | 4.89 (3.78) |
| apomorphine | N | 45 | 4.84 (3.61, 6.48) | 4.83 (3.61, 6.47) | 66.87 | 2.27 (1.73) | 4.82 (3.6) |
| deutetrabenazine | N | 39 | 9.13 (6.66, 12.5) | 9.1 (6.65, 12.45) | 190.05 | 3.18 (2.46) | 9.08 (6.63) |
| carbidopa/entacapone/levodopa | N | 37 | 10.17 (7.36, 14.05) | 10.14 (7.35, 13.98) | 216.51 | 3.34 (2.56) | 10.12 (7.33) |
| trofinetide | N | 36 | 6.91 (4.98, 9.59) | 6.9 (4.98, 9.56) | 114.67 | 2.78 (2.1) | 6.89 (4.97) |
| amitriptyline | N | 34 | 4.55 (3.25, 6.37) | 4.54 (3.25, 6.36) | 51.17 | 2.18 (1.55) | 4.54 (3.24) |
| clonidine | S | 33 | 2.94 (2.09, 4.14) | 2.94 (2.09, 4.14) | 18.73 | 1.55 (0.98) | 2.94 (2.09) |
| amantadine | N | 32 | 7.96 (5.63, 11.27) | 7.94 (5.62, 11.23) | 133.94 | 2.99 (2.21) | 7.93 (5.6) |
| galantamine | N | 31 | 9.18 (6.45, 13.07) | 9.15 (6.44, 13.01) | 163.42 | 3.19 (2.35) | 9.14 (6.42) |
| sevoflurane | N | 30 | 8.01 (5.59, 11.46) | 7.98 (5.58, 11.41) | 129.12 | 3 (2.18) | 7.97 (5.57) |
| entacapone | N | 29 | 9.8 (6.81, 14.12) | 9.77 (6.79, 14.05) | 171.5 | 3.29 (2.39) | 9.76 (6.77) |
| vigabatrin | N | 29 | 3.13 (2.18, 4.51) | 3.13 (2.18, 4.5) | 20.65 | 1.65 (1.02) | 3.13 (2.17) |
| opicapone | N | 28 | 34.4 (23.68, 49.96) | 33.94 (23.49, 49.04) | 820.64 | 5.08 (3.45) | 33.9 (23.34) |
| Articaine/epinephrine | N | 26 | 16 (10.88, 23.53) | 15.9 (10.84, 23.33) | 307.24 | 3.99 (2.8) | 15.88 (10.8) |
| levodopa | N | 26 | 3.71 (2.53, 5.46) | 3.71 (2.53, 5.45) | 28.85 | 1.89 (1.2) | 3.71 (2.52) |
| midazolam | N | 25 | 6.92 (4.68, 10.26) | 6.91 (4.67, 10.22) | 89.98 | 2.79 (1.92) | 6.9 (4.66) |
| hydroxyzine | R | 25 | 4.67 (3.16, 6.92) | 4.67 (3.15, 6.91) | 45.09 | 2.22 (1.46) | 4.66 (3.15) |
| chlorpromazine | N | 24 | 22.12 (14.8, 33.07) | 21.93 (14.72, 32.67) | 427.69 | 4.45 (3) | 21.91 (14.66) |
| lumateperone | N | 24 | 6.96 (4.66, 10.39) | 6.94 (4.65, 10.35) | 88.08 | 2.79 (1.91) | 6.94 (4.64) |
| rasagiline | J | 23 | 7.71 (5.12, 11.62) | 7.69 (5.11, 11.57) | 100.44 | 2.94 (2) | 7.69 (5.1) |
| Cilastatin/imipenem | J | 23 | 5.47 (3.63, 8.24) | 5.46 (3.63, 8.22) | 57.01 | 2.45 (1.61) | 5.46 (3.62) |
| fluphenazine | N | 21 | 31.46 (20.45, 48.4) | 31.08 (20.31, 47.55) | 569.06 | 4.96 (3.09) | 31.05 (20.19) |
| droperidol | N | 19 | 75.94 (48.1, 119.89) | 73.7 (47.32, 114.78) | 1325.92 | 6.2 (3.33) | 73.64 (46.64) |
| eletriptan | N | 19 | 5.34 (3.4, 8.38) | 5.33 (3.4, 8.36) | 47.83 | 2.41 (1.48) | 5.33 (3.4) |
| cabergoline | N | 19 | 4.8 (3.06, 7.52) | 4.79 (3.06, 7.51) | 39.48 | 2.26 (1.36) | 4.79 (3.05) |
| penicillamine | N | 18 | 29.63 (18.61, 47.17) | 29.29 (18.5, 46.37) | 460.68 | 4.87 (2.89) | 29.27 (18.38) |
| clomipramine | M | 18 | 8.73 (5.49, 13.86) | 8.7 (5.48, 13.8) | 99.68 | 3.12 (1.97) | 8.69 (5.47) |
| nortriptyline | N | 18 | 9.31 (5.86, 14.79) | 9.27 (5.85, 14.71) | 109.29 | 3.21 (2.03) | 9.27 (5.83) |
| thiethylperazine | N | 15 | 288.93 (169.2, 493.36) | 258.72 (160.26, 417.69) | 3830.24 | 8.01 (3.16) | 258.55 (151.41) |
| bromocriptine | R | 15 | 6.92 (4.17, 11.48) | 6.9 (4.16, 11.44) | 60.93 | 2.79 (1.61) | 6.9 (4.15) |
| fluvoxamine | A | 15 | 6.69 (4.03, 11.11) | 6.68 (4.03, 11.07) | 57.93 | 2.74 (1.58) | 6.67 (4.02) |
| ketamine | N | 15 | 5.89 (3.55, 9.78) | 5.88 (3.55, 9.75) | 47.29 | 2.55 (1.45) | 5.88 (3.54) |
| perphenazine | N | 14 | 38.27 (22.57, 64.88) | 37.7 (22.41, 63.4) | 480.63 | 5.24 (2.7) | 37.67 (22.22) |
| iloperidone | N | 14 | 15.29 (9.04, 25.87) | 15.2 (9.02, 25.63) | 168.58 | 3.93 (2.22) | 15.19 (8.98) |
| miglustat | A | 14 | 7.91 (4.68, 13.37) | 7.89 (4.68, 13.31) | 70.34 | 2.98 (1.69) | 7.89 (4.67) |
| buspirone | N | 14 | 5.78 (3.42, 9.77) | 5.77 (3.42, 9.73) | 43.43 | 2.53 (1.38) | 5.76 (3.41) |
| istradefylline | N | 14 | 3.63 (2.15, 6.13) | 3.62 (2.15, 6.12) | 18.57 | 1.86 (0.88) | 3.62 (2.14) |
| granisetron | A | 14 | 6.4 (3.79, 10.82) | 6.39 (3.78, 10.78) | 51.12 | 2.67 (1.49) | 6.38 (3.78) |
| cerliponase alfa | A | 13 | 30.01 (17.36, 51.86) | 29.66 (17.28, 50.92) | 343.5 | 4.89 (2.51) | 29.64 (17.15) |
| imipramine | N | 12 | 8.16 (4.63, 14.38) | 8.14 (4.62, 14.32) | 64.53 | 3.02 (1.59) | 8.13 (4.61) |
| cyclobenzaprine | M | 12 | 3.61 (2.05, 6.37) | 3.61 (2.05, 6.36) | 16.53 | 1.85 (0.79) | 3.61 (2.05) |
| pimozide | N | 11 | 41.65 (22.95, 75.6) | 40.98 (22.8, 73.64) | 416.87 | 5.36 (2.4) | 40.96 (22.57) |
| desloratadine | R | 11 | 3.65 (2.02, 6.59) | 3.64 (2.02, 6.57) | 15.77 | 1.86 (0.74) | 3.64 (2.02) |
| dexmethylphenidate | N | 10 | 4.62 (2.49, 8.6) | 4.62 (2.49, 8.58) | 22.82 | 2.21 (0.93) | 4.61 (2.48) |
| trimethoprim | J | 10 | 4.25 (2.29, 7.91) | 4.25 (2.29, 7.89) | 19.65 | 2.09 (0.84) | 4.24 (2.28) |
| zolmitriptan | N | 10 | 4.01 (2.16, 7.46) | 4.01 (2.16, 7.44) | 17.64 | 2 (0.78) | 4 (2.15) |
| dexamfetamine | N | 10 | 3.84 (2.07, 7.14) | 3.84 (2.06, 7.13) | 16.25 | 1.94 (0.74) | 3.84 (2.06) |
| olanzapine/samidorphan | N | 10 | 11.9 (6.39, 22.15) | 11.85 (6.38, 21.99) | 90.82 | 3.57 (1.7) | 11.84 (6.36) |
| viloxazine | N | 9 | 14.08 (7.31, 27.12) | 14.01 (7.3, 26.88) | 101.52 | 3.81 (1.69) | 14 (7.27) |
| isoflurane | N | 9 | 14.62 (7.59, 28.16) | 14.54 (7.58, 27.89) | 106.21 | 3.86 (1.71) | 14.53 (7.55) |
| tiotixene | N | 8 | 24.16 (12.04, 48.49) | 23.94 (12.01, 47.71) | 169.63 | 4.58 (1.79) | 23.93 (11.93) |
| palonosetron | A | 8 | 11.87 (5.93, 23.78) | 11.82 (5.92, 23.6) | 73.7 | 3.56 (1.46) | 11.81 (5.9) |
| bupropion/dextromethorphan | N | 8 | 6.27 (3.13, 12.56) | 6.26 (3.13, 12.51) | 30.96 | 2.65 (1.02) | 6.26 (3.13) |
| procyclidine | M | 7 | 68.46 (32.3, 145.1) | 66.64 (32.09, 138.4) | 447.64 | 6.06 (1.82) | 66.62 (31.43) |
| amisulpride | N | 7 | 36.24 (17.18, 76.45) | 35.73 (17.13, 74.56) | 231.43 | 5.16 (1.71) | 35.72 (16.94) |
| etomidate | N | 7 | 22.73 (10.8, 47.85) | 22.53 (10.78, 47.11) | 139.29 | 4.49 (1.58) | 22.52 (10.7) |
| benzatropine | N | 7 | 13.12 (6.24, 27.58) | 13.06 (6.24, 27.33) | 73.56 | 3.71 (1.36) | 13.05 (6.21) |
| vecuronium | N | 7 | 12.8 (6.09, 26.9) | 12.74 (6.08, 26.67) | 71.37 | 3.67 (1.34) | 12.73 (6.06) |
| suxamethonium | M | 7 | 7.81 (3.72, 16.41) | 7.79 (3.72, 16.33) | 37.68 | 2.96 (1.05) | 7.79 (3.71) |
| methylergometrine | G | 6 | 6.49 (2.91, 14.47) | 6.48 (2.91, 14.4) | 25.19 | 2.69 (0.77) | 6.48 (2.91) |
| trihexyphenidyl | N | 5 | 17.45 (7.24, 42.07) | 17.34 (7.24, 41.53) | 74.62 | 4.12 (1.04) | 17.33 (7.19) |
| dolasetron | A | 4 | 51.88 (19.27, 139.67) | 50.83 (19.27, 134.08) | 193.81 | 5.67 (0.91) | 50.82 (18.88) |
| loxapine | N | 4 | 35.59 (13.26, 95.49) | 35.09 (13.26, 92.86) | 130.89 | 5.13 (0.86) | 35.09 (13.07) |
| frovatriptan | N | 4 | 14.67 (5.49, 39.2) | 14.59 (5.49, 38.76) | 49.15 | 3.87 (0.68) | 14.58 (5.46) |
| safinamide | N | 4 | 13.13 (4.91, 35.07) | 13.06 (4.91, 34.71) | 43.1 | 3.71 (0.64) | 13.06 (4.89) |
| thioridazine | N | 4 | 11.09 (4.15, 29.61) | 11.04 (4.15, 29.36) | 35.13 | 3.46 (0.58) | 11.04 (4.13) |
| tranylcypromine | N | 4 | 5.51 (2.07, 14.71) | 5.5 (2.07, 14.65) | 13.64 | 2.46 (0.24) | 5.5 (2.06) |
| molindone | A | 3 | 121.19 (38.02, 386.27) | 115.55 (38.28, 348.83) | 339.89 | 6.85 (0.48) | 115.54 (36.25) |
| domperidone | A | 3 | 15.63 (5.02, 48.64) | 15.54 (5.03, 48.01) | 39.96 | 3.96 (0.3) | 15.53 (4.99) |
| nabilone | A | 3 | 13.37 (4.3, 41.58) | 13.3 (4.3, 41.12) | 33.31 | 3.73 (0.26) | 13.3 (4.28) |
| trientine | N | 3 | 11.3 (3.64, 35.14) | 11.26 (3.64, 34.81) | 27.24 | 3.49 (0.21) | 11.25 (3.62) |
| pentazocine | N | 3 | 10.24 (3.29, 31.82) | 10.2 (3.3, 31.56) | 24.12 | 3.35 (0.18) | 10.2 (3.28) |
| dexmethylphenidate/serdexmethylphenidate | N | 3 | 8.62 (2.77, 26.77) | 8.59 (2.78, 26.58) | 19.37 | 3.1 (0.12) | 8.59 (2.76) |

*Note1: ranked by case reports*

*Note2: Signals are detected when all the following criteria are met: a ≥ 3, PRR ≥2 and Chi-Square ≥ 4, lower limit of 95% CI of ROR > 1, IC_025_ > 0, EBGM_05_ > 2*


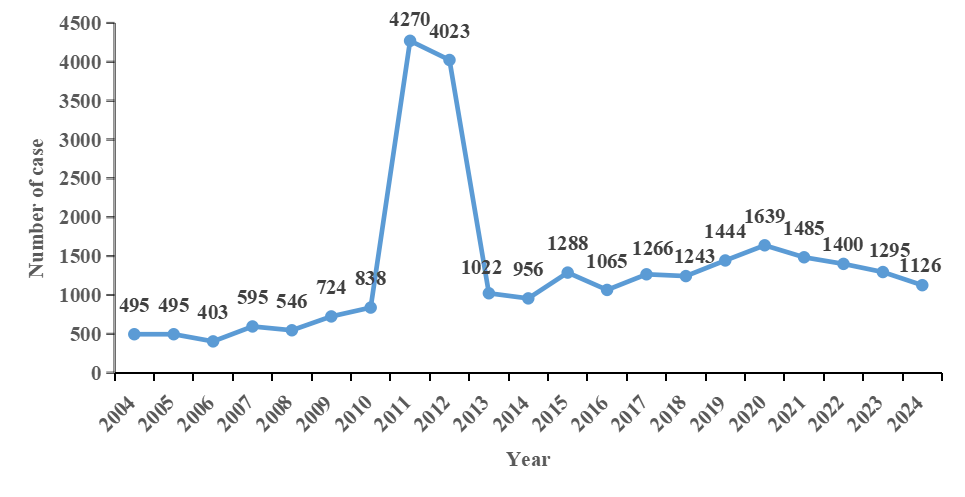


**Supplementary Fig.1.** Number of reported cases of dystonia from the first quarter of 2004 to the third quarter of 2024
